# 

**Published:** 1893-01

**Authors:** 


### Society Meetings.

The Medical Society of the County of Erie will hold its annual meeting on Tuesday, January 10, 1893, in the Y. M. C. A. building, corner West Mohawk, Pearl, and West Genesee streets, beginning at ten o'clock A. M.

The following programme is offered for the entertainment of the society :

I. Discussion of Hydatid Disease :

Pathology. By Dr. Roswell Park.

Symptomatology and Diagnosis. By Dr. J. H. Pryor.

Surgery. By Dr. Herman Mynter.

II. The Relation of Gonorrhea to Pelvic Diseases in Women. By Drs. M. D. Mann, C. C. Frederick, and M. A. Crockett.

The sixth annual meeting of the National Association of Railway Surgeons, embracing the United States, Canada, and Mexico, will be held at Omaha, Neb., the last Wednesday, Thursday, and Friday of May, 1893. A preliminary announcement has been issued, which embraces thirteen different titles besides one general subject for discussion, namely, Injuries of the Cord and its Envelopes without Fracture of the Spine. Dr. C. W. P. Brock, of Richmond, Va., is President, and Dr. E. R. Lewis, of Kansas City, Mo., is Secretary.

The Medical Society of the State of New York will hold its eighty-seventh annual meeting in the City Hall, at Albany, on Tuesday, Wednesday, and Thursday, February 7, 8, and 9, 1893. All members of the profession are cordially invited to attend.

### BOOKS RECEIVED.

Text-Book of Ophthalmology. By Dr. Ernest Fuchs, Professor of Ophthalmology in the University of Vienna. Authorized translation from the second enlarged and improved German edition. By A. Duane, M. D., Assistant Surgeon Ophthalmic and Aural Institute, New York. With numerous illustrations. Octavo, pp. xiv.—188. New York: D. Appleton & Co. 1892.

A Treatise on Diseases of the Rectum, Anus, and Sigmoid Flexure. By Joseph M. Mathews, M. D., Professor of Principles and Practice of Surgery, and Clinical Lecturer on Diseases of the Rectum, Kentucky School of Medicine; Visiting Surgeon, St. Mary and Elizabeth Hospital; Consulting Surgeon, Louisville City Hospital; Consulting Surgeon, Jennie Cassady Free Infirmary for Women; late President Mississippi Valley Medical Association; President Louisville Clinical Society; Vice-President Louisville Surgical Society; Member International Medical Congress, American Medical Association, Southern Surgical and Gynecological Society, Kentucky State Medical Society, State Board of Health of Kentucky; Orator of the American Medical Association on Surgery, 1891, etc. With six chromo-lithographs and numerous illustrations. Octavo, pp. xvi.—537. New York: D. Appleton & Co. 1892.

Diseases of the Eye, Ear, Throat, and Nose. A Manual for Students and Practitioners. By Frank E. Miller, M. D., Attending Physician, St. Joseph's Hospital; Throat Surgeon, Vanderbilt Clinic, New York; James P. McEvoy, M. D., Throat Surgeon, Bellevue Hospital, Out-Patient Department, New York; and John E. Weeks, M. D., Surgeon, New York Eye and Ear Infirmary; Lecturer on Ophthalmology and Otology, Bellevue Hospital Medical College, New York.

The Students' Quiz Series. Physiology, by Frederick A. Manning, M. D., Attending Surgeon, Manhattan Hospital, New York. Series edited by Bern B. Gallaudet, M. D., Demonstrator of Anatomy, College of Physicians and Surgeons, New York. Visiting Surgeon, Bellevue Hospital, New York. Pocket size, 12mo, 201 pages, sixty-nine illustrations, $1.00. Philadelphia: Lea Brothers & Co.

A Treatise on Nervous and Mental Diseases. By Landon Carter Gray, M. D., Professor of Nervous and Mental Diseases in the New York Polyclinic; Ex-President of the American Neurological Association, etc. In one very handsome octavo volume of 681 pages, with 168 fine engravings. Cloth, $4.50; leather, $5.50. Philadelphia: Lea Brothers & Co. 1893.

A Manual of Bacteriology. By George M. Sternberg, M. D., Deputy Surgeon-General, U. S. Army; Director of the Hoagland Laboratory, Brooklyn, N. Y.; Honorary Member of the Epidemiological Society, of London; of the Royal Academy of Medicine, of Rome; of the Academy of Medicine, of Rio de Janeiro; of the American Academy of Medicine, etc., etc. Illustrated by heliotype and chromo-lithographic plates, and 268 engravings. New York: William Wood & Co. 1892.

Handbook of Pathological Anatomy and Histology, with an introductory section on Post-Mortem Examinations and the Methods of Preserving and Examining Diseased Tissues. By Francis Delafield, M. D., LL. D., Professor of the Practice of Medicine, College of Physicians and Surgeons, Columbia College, New York, and T. Mitchell Prudden, M. D., Professor of Pathology and Director of the Laboratories of Histology, Pathology, and Bacteriology, College of Physicians and Surgeons, Columbia College, New York. Illustrated by 300 wood engravings printed in black and colors. Fourth revised and enlarged edition. One large octavo volume of 732 pages, with 300 wood engravings, beautifully printed on fine super paper, and bound in blue imported muslin. Price, $6.00. New York: William Wood Co. 1892.

Transactions of the American Surgical Association. Volume the tenth. Edited by J. Ewing Mears, M. D., Recorder of the Association. Philadelphia: Printed for the Association, and for sale, by Wm. J. Dornan. 1892.

A Manual of Physics; being an Introduction of the Study of Physical Science. Designed for the use of University students. By William Peddie, D. Sc., F. R. S. R. Assistant to the Professor of Natural Philosophy in the University of Edinburgh. New York: G. P. Putnam's Son. London: Baillière, Tindall & Cox. 1892.

The Anatomy of the Perineum. By Franklin Dexter, M. D., Assistant Demonstrator of Anatomy, College of Physicians and Surgeons, Columbia University, New York. With thirty-eight illustrations. New York: D. Appleton & Co. 1892.

Handbook of Physiology. By W. Morrant Baker, F. R. C. S., Late Surgeon to and Lecturer on Physiology at St. Bartholomew's Hospital, etc., and Vincent Dormer Harris, M. D., London, Examiner of Physiology at the Conjoint Board of the Royal College of Physicians and Surgeons, and in the University of Durham; Demonstrator of Physiology at St. Bartholomew's Hospital; Physician to the Victoria Park Hospital for Diseases of the Chest. Thirteenth edition. With upwards of 500 illustrations, including some colored plates. Philadelphia: P. Blakiston, Son & Co., 1012 Walnut street. 1892.

Addresses and Essays. By G. Frank Lydston, M. D., Professor of the Surgical Diseases of the Genito-Urinary Organs and Syphology, in the Chicago College of Physicians and Surgeons; Surgeon-in-Chief of the Genito-Urinary and Venereal Department of the West Side Dispensary, Chicago; Fellow of the Chicago Academy of Medicine, and of the Southern Surgical and Gynecological Association; Lecturer on Criminal Anthropology in the Union Law School; Honorary Member of the Texas State Medical Association. Second edition, revised and enlarged. Published by Renz & Henry, Louisville, Ky.

Acne and Alopecia. By L. Duncan Bulkley, A. M., M. D., Professor of Diseases of the Skin, New York Post-Graduate Medical School; Physician to the New York Skin and Cancer Hospital, etc. Geo. S. Davis, Detroit, Mich. 1892.

Diseases of the Lungs, Heart, and Kidneys. By N. S. Davis, Jr., A. M., M. D., Professor of Principles and Practice of Medicine, Chicago Medical College; Physician to Mercy Hospital; Member of the American Medical Association, Illinois State Medical Society, Chicago Medical Society, Chicago Academy of Sciences, Illinois State Microscopical Society; Fellow of the American Academy of Medicine; Author of Consumption, How to Prevent It, and How to Live with It," etc. No. 14 in the Physicians' and Students' Ready-Reference Series. In one neat 12mo volume of 359 pages, extra cloth, $1.25 net. Philadelphia: The F. A. Davis Co., 1231 Filbert street.

The Students' Quiz Series. Edited by Bern B. Gallaudet, M. D., Demonstrator of Surgery, College of Physicians and Surgeons, New York. Volume XIII. Diseases of Children. By C. A. Rhodes, M. D., Instructor in Diseases of Children, New York Post-Graduate Medical School. Pocket size, 12mo, 170 pages. Limp cloth, $1.00. Philadelphia: Lea Brothers & Co., 1892.

Human Embryology, By Charles Segwick Minot, Professor of Histology and Human Embryology, Harvard Medical School, Boston. With 463 illustrations. New York: William Wood & Co. 1892.

---

Notice to Contributors.—We are glad to receive contributions from every one who knows anything of interest to the profession. Articles designed for publication in the Journal should be handed in before the first day of the month. The Editors are not responsible for the views or opinions of contributors. All communications should be addressed to the Managing Editor, 284 Franklin St., Buffalo, N. Y.

